# Enhancing endogenous levels of GLP1 dampens acute olanzapine induced perturbations in lipid and glucose metabolism

**DOI:** 10.3389/fphar.2023.1127634

**Published:** 2023-03-01

**Authors:** Kyle D. Medak, Alyssa J. Weber, Hesham Shamshoum, Greg L. McKie, Margaret K. Hahn, David C. Wright

**Affiliations:** ^1^ Department of Human Health and Nutritional Science, University of Guelph, Guelph, ON, Canada; ^2^ Centre for Addition and Mental Health, Toronto, ON, Canada; ^3^ Institute of Medical Science, Faculty of Medicine, University of Toronto, Toronto, ON, Canada; ^4^ Department of Psychiatry, University of Toronto, Toronto, ON, Canada; ^5^ Banting and Best Diabetes Centre, University of Toronto, Toronto, ON, Canada; ^6^ Department of Pharmacology and Toxicology, University of Toronto, Toronto, ON, Canada; ^7^ School of Kinesiology, University of British Columbia, Vancouver, BC, Canada; ^8^ Faculty of Food and Land Systems, University of British Columbia, Vancouver, BC, Canada; ^9^ BC Children’s Hospital Research Institute, Vancouver, BC, Canada

**Keywords:** antipsychotics, glucagon, mice, Glp1, glucose, insulin

## Abstract

Olanzapine is a second-generation antipsychotic (SGA) used in the treatment of schizophrenia and several on- and off-label conditions. While effective in reducing psychoses, acute olanzapine treatment causes rapid hyperglycemia, insulin resistance, and dyslipidemia and these perturbations are linked to an increased risk of developing cardiometabolic disease. Pharmacological agonists of the glucagon-like peptide-1 (GLP1) receptor have been shown to offset weight-gain associated with chronic SGA administration and mitigate the acute metabolic side effects of SGAs. The purpose of this study was to determine if increasing endogenous GLP1 is sufficient to protect against acute olanzapine-induced impairments in glucose and lipid homeostasis. Male C57BL/6J mice were treated with olanzapine, in the absence or presence of an oral glucose tolerance test (OGTT), and a combination of compounds to increase endogenous GLP1. These include the non-nutritive sweetener allulose which acts to induce GLP1 secretion but not other incretins, the DPPiv inhibitor sitagliptin which prevents degradation of active GLP1, and an SSTR5 antagonist which relieves inhibition on GLP1 secretion. We hypothesized that this cocktail of agents would increase circulating GLP1 to supraphysiological concentrations and would protect against olanzapine-induced perturbations in glucose and lipid homeostasis. We found that ‘triple treatment’ increased both active and total GLP1 and protected against olanzapine-induced perturbations in lipid and glucose metabolism under glucose stimulated conditions and this was paralleled by an attenuation in the olanzapine induced increase in the glucagon:insulin ratio. Our findings provide evidence that pharmacological approaches to increase endogenous GLP1 could be a useful adjunct approach to reduce acute olanzapine-induced perturbations in lipid and glucose metabolism.

## Graphical Abstract

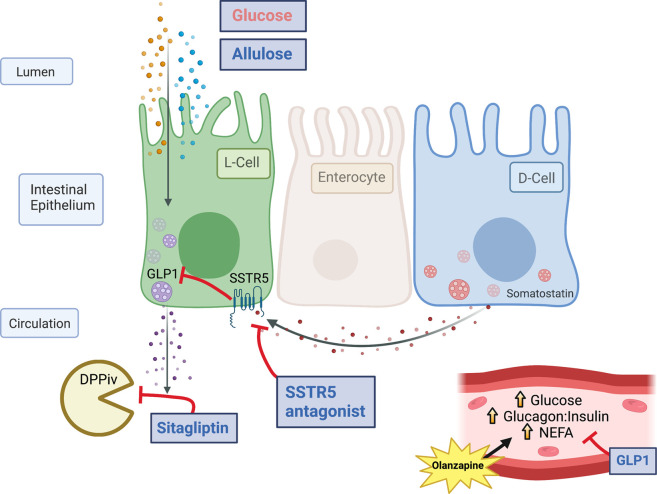

## Introduction

Schizophrenia imposes a disproportionately large economic burden relative to other mental illnesses and non-psychiatric medical disorders (Rupp and Keith, 1993). Antipsychotic drugs (APs) such as olanzapine are commonly used in the treatment of schizophrenia and in a wide list of on- and off-label conditions such as anxiety, sleep disorders, dementia, bipolar, and attention deficit disorder, to name a few (Maher et al., 2011). Olanzapine lessens symptoms of psychosis through action on the dopamine (D_2_), serotonin (5-HT_2A_) and muscarinic (M_3_) receptors (Divac et al., 2014) but is also associated with severe peripheral metabolic consequences including hyperglycemia, insulin resistance, hyperlipidemia, weight gain and the development of type 2 diabetes all of which lead to increases in mortality [ (De Hert et al., 2009; Rojo et al., 2015; Castellani et al., 2019; Huhn et al., 2019; Kowalchuk et al., 2019; Barton et al., 2020; Pillinger et al., 2020)].

The metabolic complications induced by APs had initially been attributed to weight gain, which is a common side effect and key co-determinant in AP-induced metabolic dysfunction (Townsend et al., 2018). Contrary to this assertation, acute treatments in preclinical models [ (Bush et al., 2018; Castellani et al., 2018; Medak et al., 2019; Shamshoum et al., 2019; Medak et al., 2020; Shamshoum et al., 2021a; Shamshoum et al., 2022)] and in human participants (Albaugh et al., 2011a), (Hahn et al., 2013), have demonstrated profound impairments in glucose metabolism within minutes to hours, highlighting weight gain independent effects of APs. Acute excursions in blood glucose, as would be observed with each treatment of olanzapine, can be harmful as they increase the risk for the development of oxidative stress, inflammation, and cardio-metabolic disease (Chiasson et al., 2002), (Chiasson et al., 2003). Co-treatment strategies which lessen the acute metabolic impairment induced by APs are thus warranted.

Our group has provided evidence that increases in glucagon mediate acute olanzapine-induced hyperglycemia. In support of this, our group and others have shown that 1) acute treatment with olanzapine increases circulating glucagon (Townsend et al., 2018), (Shamshoum et al., 2019), (Shamshoum et al., 2022), (Shamshoum et al., 2021a), 2) the hyperglycemic effects of olanzapine are absent in glucagon receptor knockout mice (Castellani et al., 2017), 3) protection against olanzapine-induced hyperglycemia is often paralleled by reductions in glucagon and/or the glucagon:insulin ratio (Medak et al., 2019), (Shamshoum et al., 2019), and 4) suppressing pancreatic secretions with somatostatin blocked olanzapine-induced hyperglycemia (Castellani et al., 2022).

GLP1 is a hormone which can reduce the glucagon:insulin ratio and is higher in female mice, a model of protection from olanzapine-induced hyperglycemia, compared to male or ovariectomized mice (Medak et al., 2019), (Handgraaf et al., 2018). In recent work we have shown that the pharmacological activation of the glucagon-like peptide-1 (GLP1) receptor with agonists such as liraglutide and exendin 4 protect against olanzapine induced increases in glucagon and hyperglycemia, while antagonizing the GLP1 receptor potentiates the blood glucose response to olanzapine (Medak et al., 2020). Collectively, these data provide evidence demonstrating that targeting the GLP1 receptor is an efficacious approach to limit the acute metabolic perturbations of olanzapine. Unfortunately, injectable agents are associated with adverse effects in patients (Harris and McCarty, 2015). Currently, it is not known if increasing circulating GLP1 using oral compounds would confer the same protective effects against olanzapine-induced hyperglycemia as GLP1 receptor agonism. In the current study we sought to test this premise by cotreating mice with olanzapine and a variety of compounds either alone or in combination which increase endogenous GLP1 concentrations including: the non-nutritive sweetener Allulose (or D-psicose) which acts to induce GLP1 secretion but not other incretins (Hayakawa et al., 2018), (Iwasaki et al., 2018), the dipeptidyl peptidase-4 (DPPiv) inhibitor Sitagliptin which prevents degradation of the active form of GLP1 (Doustmohammadian et al., 2022), and a somatostatin receptor 5 (SSTR5) antagonist to relieve inhibition on GLP1 secretion (Liu et al., 2018a). A similar cocktail of agents has been shown to markedly increase circulating GLP1 and to improve glucose tolerance in mice (Briere et al., 2018). We hypothesized that ‘triple treatment’ with agents which stimulated GLP1 secretion and protected against GLP1 degradation would increase endogenous GLP1 to supraphysiological concentrations (Briere et al., 2018) and protect against acute olanzapine-induced perturbations in glucose and lipid metabolism.

## Materials and methods

### Animals

All experimental procedures were approved by the University of Guelph Animal Care Committee and followed Canadian Council on Animal Care guidelines. Approximately 10-week-old male C57BL/6J mice were purchased from Jackson Laboratories (Bar Harbor, ME) and individually housed in clear polycarbonate shoebox-style cages (dimensions: 7 1⁄2″ x 11 1⁄2″ x 5”) with wire lids. We used only male mice in these experiments as female mice are already protected against olanzapine-induced hyperglycemia (Medak et al., 2019). Rooms were kept at an ambient temperature of 22 °C with 45% humidity and a 12:12 h light dark cycle. Animals were given free access to water and standard rodent chow (7004-Teklad S-2335 Mouse Breeder Sterilizable Diet; Teklad Diets Harlan Laboratories, Madison WI). Mice were acclimated to our facilities for ∼10 days before experimentation. All olanzapine experiments occurred at the beginning of the animals’ light cycle which coincides with the clinical recommendation for drug administration prior to bedtime (Miller, 2004).

### Materials

Olanzapine (cat. 11937) was purchased from Cayman Chemicals (Ann Arbor, MI, United States). Dimethyl sulfoxide (DMSO) was from Wako Pure Chemical Industries (cat. 67-68-5; Richmond, VA, United States). Kolliphor EL was from Millipore Sigma (Etobicoke, ON, CA; cat. C5135). Allulose (cat. P839620-1), Sitagliptin (cat. 13252), Captisol (cat. S4592), and SSTR5 antagonist (cat. HY-1021037) were purchased from Cedarlane (Burlington, ON, CA). Blood glucose test strips and a Freestyle Lite handheld glucometer were acquired from Abbott Diabetes Care Inc. (Alameda, CA, United States). Injections were carried out using 25-gauge needles purchased from ThermoFisher Scientific (Mississauga, ON, CAN; cat. BD B305122) and 29-gauge insulin needles purchased from VWR (Radnor, PA, United States; cat. 10799-004). ELISAs obtained from Mercodia Inc. (Winston-Salem, NC 27103, United States) were used to measure serum glucagon (cat. 10-1281-01) and insulin (cat. 10-1247-01). Colorimetric assays used to measure Beta-Hydroxybutyrate (cat. 700190) and serum Triglyceride (cat. 10010303) were obtained from Cayman Chemicals (Ann Arbor, MI, United States). ELISAs used to measure total GLP1 (cat. EZGLP1T-36K) and active GLP1 (cat. EGLP-35K) were obtained from Sigma Aldrich (St. Louis, MO, United States). Serum non-esterified fatty acid (NEFA) (Wako Bioproducts, Richmond, VA, United States) and glycerol (F6428; Millipore Sigma, St. Louis, MO, United States) were measured on 96-well plates as our group has previously described (Snook et al., 2016) and as suggested by manufacturer instruction.

### Terminal olanzapine tolerance test

Olanzapine was dissolved in DMSO (1 mg/100 μL) to create a stock solution. Kolliphor EL solution and saline (500 μL/900 mL) were used to dilute 500 μL of the stock olanzapine solution and mice were injected intraperitoneally (IP) with olanzapine (5 mg/kg) or vehicle (DMSO, Kolliphor EL, saline) at the beginning of the light cycle (∼0900). Drug and vehicle were prepared from powdered drug and stored stock solutions (DMSO, Kolliphor EL, and saline) each experimental day. We (Townsend et al., 2018), (Castellani et al., 2017) and others (Ikegami et al., 2013a) have previously used this dose of olanzapine as it mimics human dosing requirements based on dopamine-binding occupancy in rats given olanzapine by subcutaneous injections (Kapur et al., 2003). Allulose was dissolved in water and orally gavaged (1 g/kg). Sitagliptin was dissolved in phosphate-buffered saline (10 mg/kg) and gavaged as a pre-treatment, 30 min before olanzapine treatment. SSTR5 antagonist was dissolved with 30% captisol and saline (15 mg/kg). Blood glucose was measured in mice prior to, 15-, 30-, 60-, 90-, and 120-min post-drug or control administration using a handheld glucometer sampled from a drop of blood taken from the tail vein using a distal tail snip. In a separate cohort of animals, the combination treatment (“triple treatment”) experiment was repeated under glucose stimulated conditions (0.5 g/kg) in which glucose was added to the gavage containing allulose or water and gavaged at the same time as olanzapine treatment. Mice were not fasted prior to the oral glucose tolerance test as this intervention itself ablates the hyperglycemic effect of olanzapine (Shamshoum et al., 2022). At 120 min post treatment mice were anesthetized with sodium pentobarbital (5 mg/100 g body weight) then cardiac blood was collected with 25-gauge needles, allowed to clot for ∼20 min at room temperature, and centrifuged at 5000 *g* for 10 min at 4°C. Tissues were stored at −80°C until further analysis.

### Serum olanzapine and N-desmethyl-olanzapine measurements

Serum samples (∼500 μL) were collected, and concentrations of olanzapine and the metabolite N-desmethyl-olanzapine (DMO), were assayed using liquid chromatography with tandem mass spectrometry detection (Graff-Guerrero et al., 2015).

### Statistical analyses

Statistical tests were completed using GraphPad Prism v.9.0 (GraphPad Software, La Jolla, CA, USA). Comparison of two groups was done by an unpaired, 2-tailed *t*-test while the effects of drug co-treatment on glucose AUC and serum measures were analyzed by two-way ANOVA. If a significant interaction was detected a Tukey’s post-hoc analysis was completed in which case the *p-value* of the discussed group comparison is represented (*p < 0.05, **p < 0.01, ***p < 0.001, ****p < 0.0001 between indicated groups). Data sets were analyzed for outliers with Grubbs’ test using Graphpad Outlier Calculator and values were excluded if identified as outliers. Significant main effects are indicated by a line above the graph while bars connected by lines indicate a significant difference between groups. Glucose curves were displayed but not statistically analyzed as this information was represented in AUC values. Normality was assessed using the Shapiro-Wilk test unless a sample size was large enough to use the D’Agostino & Pearson test, as per the recommendation of Graphpad Statistics Guide. A relationship was considered significant when *p* < 0.05.

## Results

### Allulose increases total serum GLP1 but is not sufficient to protect against acute olanzapine-induced metabolic disturbances

**FIGURE 1:**
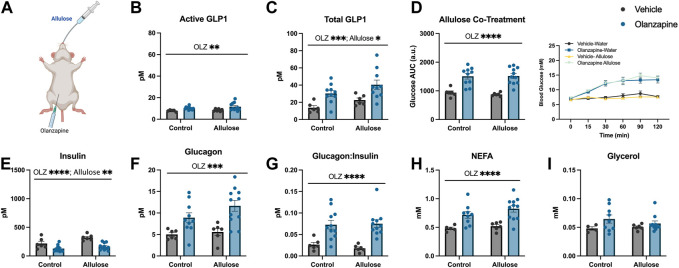
Allulose cotreatment does not protect against olanzapine-induced changes in glucose homeostasis. Mice were co-treated with an IP injection of olanzapine (5 mg/kg) and allulose gavage (1 g/kg) **(A)** for 120 min (*n* = 6–11 mice/group). **(B, C)** Active and total GLP1 were measured from serum following 120 min from injection. **(D)** Blood glucose was measured from the distal tail blood and area under the curve (AUC) calculated. In serum collected from cardiac blood, insulin **(E)**, glucagon **(F)**, the ratio of glucagon:insulin **(G)**, Non-Esterified Fatty Acids **(H)**, and glycerol **(I)** were measured. Blood glucose AUC and serum hormones/metabolites were analyzed by two-way ANOVA. Lines over graphs indicate a main effect of the described parameter. *p < 0.05, **p < 0.01, ***p < 0.001, ****p < 0.0001 between indicated groups. All data are presented as mean ± SEM.

As a follow-up to our previous study in which GLP1 receptor agonists fully protected against olanzapine-induced hyperglycemia and markers of lipid dysregulation (Medak et al., 2020) we aimed to determine if increasing endogenous GLP1 could be protective in the same way. As a first approach we utilized allulose (1 g/kg, gavage) as a GLP1 secretagogue (Hayakawa et al., 2018) to co-treat with olanzapine (5 mg/kg, IP) (Figure 1A). In serum, active GLP1 measured 120-min post-treatment was increased by olanzapine (Mean ± SD: Vehicle-Control = 7.86 ± 0.77, Olanzapine-Control = 10.16 ± 1.41, Vehicle-Allulose = 8.48 ± 1.19, Olanzapine-Allulose = 11.61 ± 3.65; Figure 1B) while there were main effects of both allulose and olanzapine to increase total GLP1 (Mean ± SD: Vehicle-Control = 13.72 ± 5.77, Olanzapine-Control = 30.26 ± 10.86, Vehicle-Allulose = 22.63 ± 5.89, Olanzapine-Allulose = 40.43 ± 17.09; Figure 1C). The minor increase in serum GLP1 did not impact olanzapine induced increases in blood glucose where there was a main effect of olanzapine to increase the glucose AUC (Figure 1D). In line with this finding, there were main effects of olanzapine to reduce insulin (Figure 1E) and increase glucagon (Figure 1F) and the ratio of glucagon:insulin (Figure 1G).

Consistent with prior work from our group (Medak et al., 2019)– (Medak et al., 2020), (Shamshoum et al., 2021a), (Shamshoum et al., 2022), there was a main effect of olanzapine to increase serum non-esterified fatty acids (NEFA) (Figure 1H), while circulating glycerol concentrations were unchanged with treatment (Figure 1I). Taken together, the data from this experiment demonstrates that marginally increasing total GLP1 is not sufficient to protect against olanzapine-induced metabolic disturbances and that more robust increases in total and/or active GLP1 may be required to blunt the metabolic consequences of acute olanzapine treatment.

### Sitagliptin is not sufficient to protect against acute olanzapine-induced metabolic disturbances

**FIGURE 2:**
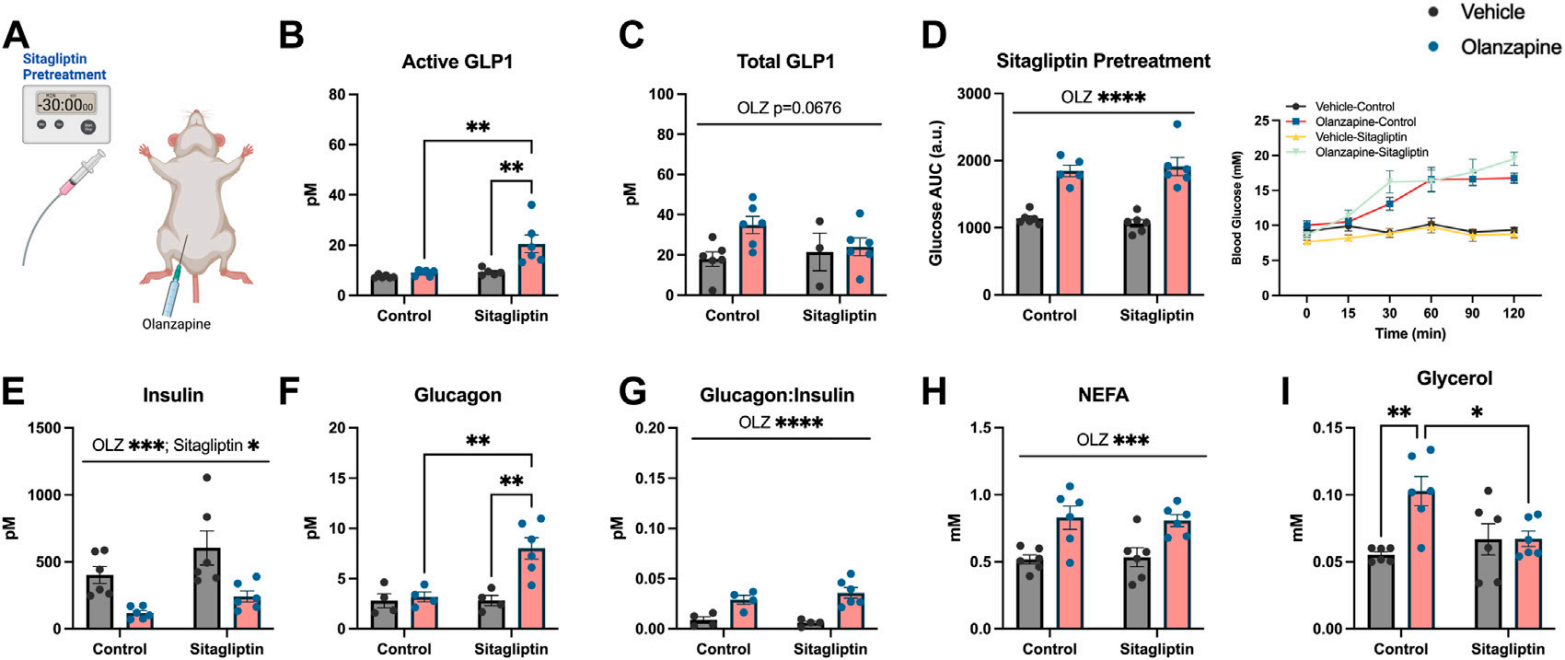
Sitagliptin pretreatment does not protect against olanzapine-induced changes in glucose homeostasis. Mice were treated with oral gavage of sitagliptin (10 mg/kg) 30 min prior to an IP injection of olanzapine (5 mg/kg) **(A)** (*n* = 4-6 mice/group). **(B, C)** Active and total GLP1 were measured from serum following 120 min from injection. **(D)** Blood glucose was measured from the distal tail blood and area under the curve (AUC) calculated. In serum collected from cardiac blood, insulin **(E)**, glucagon **(F)**, the ratio of glucagon:insulin **(G)**, Non-Esterified Fatty Acids **(H)**, and glycerol **(I)** were measured. Blood glucose AUC and serum hormones/metabolites were analyzed by two-way ANOVA. Lines over graphs indicate a main effect of the described parameter while bars connected by lines indicate a significant difference between indicated groups. *p < 0.05, **p < 0.01, ***p < 0.001, ****p < 0.0001. All data are presented as mean ± SEM.

As slight increases in total GLP1 were not sufficient to protect against olanzapine-induced excursions in blood glucose we reasoned that pharmacologically increasing the active form of this incretin peptide might confer protection against olanzapine. To do this we pretreated mice with sitagliptin (10 mg/kg), 30 min prior to olanzapine treatment (Figure 2A) and tracked changes in blood glucose. Sitagliptin is an inhibitor of DPPiv which degrades the active form of GLP1 (Drucker, 2021). Pretreatment with sitagliptin increased active (Mean ± SD: Vehicle-Control = 7.50 ± 0.62, Olanzapine-Control = 9.01 ± 1.15, Vehicle-Sitagliptin = 9.31 ± 1.38, Olanzapine-Sitagliptin = 20.53 ± 8.64; Figure 2B), but not total (Mean ± SD: Vehicle-Control = 17.96 ± 8.81, Olanzapine-Control = 34.90 ± 10.37, Vehicle-Sitagliptin = 21.51 ± 16.27, Olanzapine-Sitagliptin = 24.04 ± 10.72; Figure 2C) GLP1 under olanzapine stimulated conditions. Despite an ∼ 2-fold increase in active GLP1, olanzapine-induced hyperglycemia (Figure 2D), and reductions in serum insulin (Figure 2E) were unchanged by sitagliptin, while glucagon was elevated in animals that received both drugs (Figure 2F). The ratio of glucagon:insulin (Figure 2G) and serum NEFA (Figure 2H) were increased by olanzapine and unchanged by sitagliptin. Olanzapine increased serum glycerol in vehicle treated mice and this was attenuated in mice pre-treated with sitagliptin (Figure 2I). Collectively, this experiment demonstrates that increasing active GLP1 through the pharmacological inhibition of DPPiv is not sufficient to mute the effects of olanzapine on the development of hyperglycemia.

### Triple treatment with allulose, sitagliptin, and sstr5 antagonist did not protect against acute olanzapine-induced changes in glucose and lipid metabolism

**FIGURE 3:**
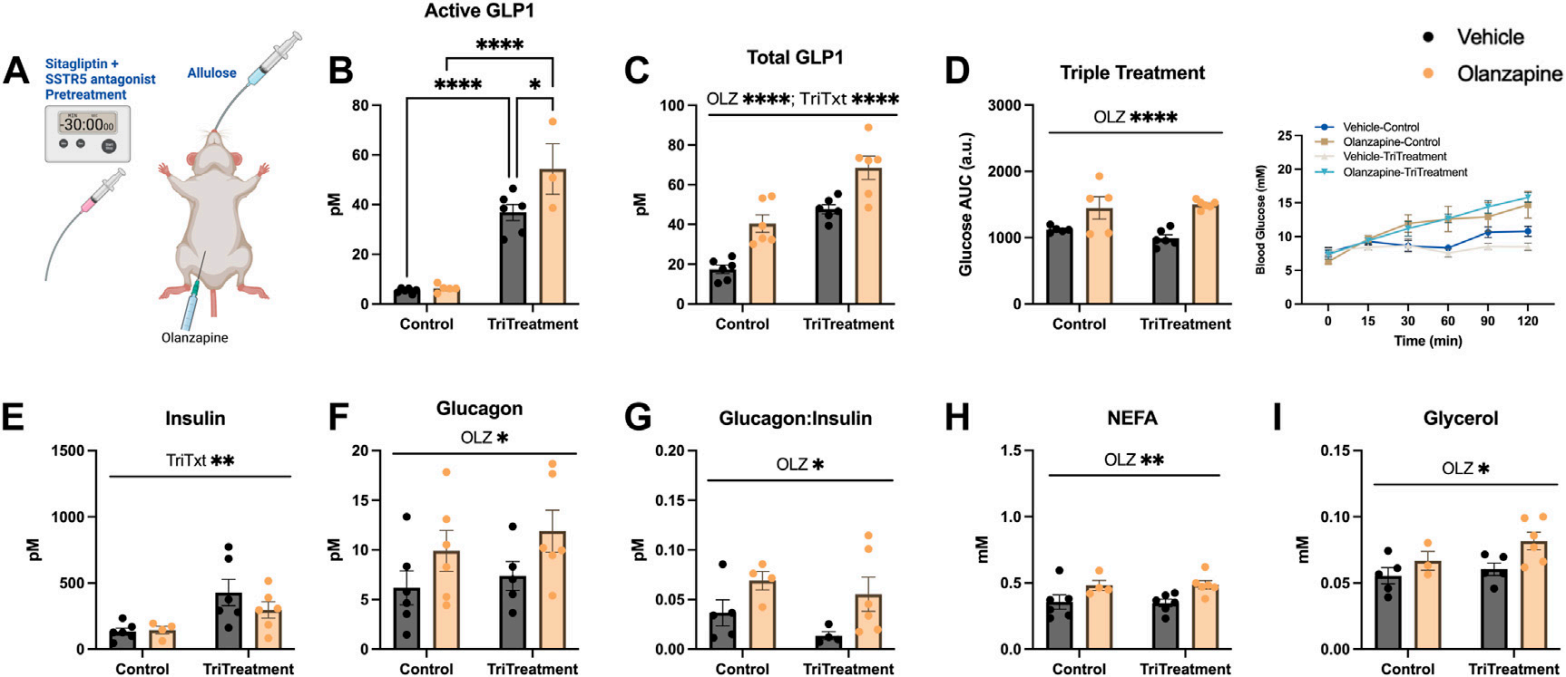
Triple treatment with allulose, sitagliptin, and sstr5 antagonist does not protect against olanzapine-induced changes in glucose or lipid homeostasis. Mice were treated with a mixed oral gavage of sitagliptin (10 mg/kg) and SSTR5 antagonist (15 mg/kg) 30 min prior to an oral gavage of allulose (1 g/kg) and an IP injection of olanzapine (5 mg/kg) **(A)** (*n* = 4–6 mice/group). **(B, C)** Active and total GLP1 were measured from serum following 120 min from injection. **(D)** Blood glucose was measured from the distal tail blood and area under the curve (AUC) calculated. In serum collected from cardiac blood, insulin **(E)**, glucagon **(F)**, the ratio of glucagon:insulin **(G)**, Non-Esterified Fatty Acids **(H)**, and glycerol **(I)** were measured. Blood glucose AUC and serum hormones/metabolites were analyzed by two-way ANOVA. Lines over graphs indicate a main effect of the described parameter while bars connected by lines indicate a significant difference between indicated groups. *p < 0.05, **p < 0.01, ***p < 0.001, ****p < 0.0001. All data are presented as mean ± SEM.

As either increasing total GLP1 or active GLP1 were not sufficient to protect against olanzapine induced hyperglycemia we reasoned an approach to increase endogenous GLP1 higher might confer protection against olanzapine induced increases in blood glucose. In this light we used a triple treatment of compounds to elevate GLP1. This included allulose (1 g/kg), sitagliptin (10 mg/kg), and SSTR5 antagonist (15 mg/kg) to induce secretion of endogenous GLP1 (Hayakawa et al., 2018), (Iwasaki et al., 2018), prevent the degradation of active GLP1 (Doustmohammadian et al., 2022), and relieve inhibition on GLP1 secretion (Liu et al., 2018a), (Jepsen et al., 2021), (Liu et al., 2018b), respectively. A solution of sitagliptin and SSTR5 antagonist was gavaged 30 min prior to olanzapine treatment while allulose treatment occurred at the same time as olanzapine (Figure 3A). When measured 120 min after olanzapine, our triple treatment significantly increased active GLP1 in serum (Mean ± SD: Vehicle-Control = 5.36 ± 0.99, Olanzapine-Control = 6.33 ± 1.53, Vehicle-TriTreatment = 36.81 ± 7.93, Olanzapine-TriTreatment = 54.29 ± 17.67; Figure 3B) with the largest increase in animals that received triple treatment and olanzapine. Total GLP1 was higher as shown by a significant main effect of both olanzapine and triple treatment (Mean ± SD: Vehicle-Control = 17.46 ± 5.33, Olanzapine-Control = 40.37 ± 10.76, Vehicle-TriTreatment = 47.67 ± 5.63, Olanzapine-TriTreatment = 68.52 ± 14.44; Figure 3C). Surprisingly, despite these large increases in GLP1, triple treatment did not protect against olanzapine-induced increases in blood glucose (Figure 3D), though there was a significant main effect of treatment to increase insulin (Figure 3E). Triple treatment did not blunt olanzapine induced increases in glucagon (Figure 3F), glucagon:insulin (Figure 3G), NEFA (Figure 3H), or glycerol (Figure 3I). In this experiment we demonstrate that increasing serum GLP1 concentrations through a combination of stimulating secretion and preventing degradation does not confer protection against acute olanzapine-induced perturbations in glucose and lipid metabolism.

### Triple treatment during OGTT improved acute olanzapine-induced changes in glucose and lipid homeostasis

**FIGURE 4:**
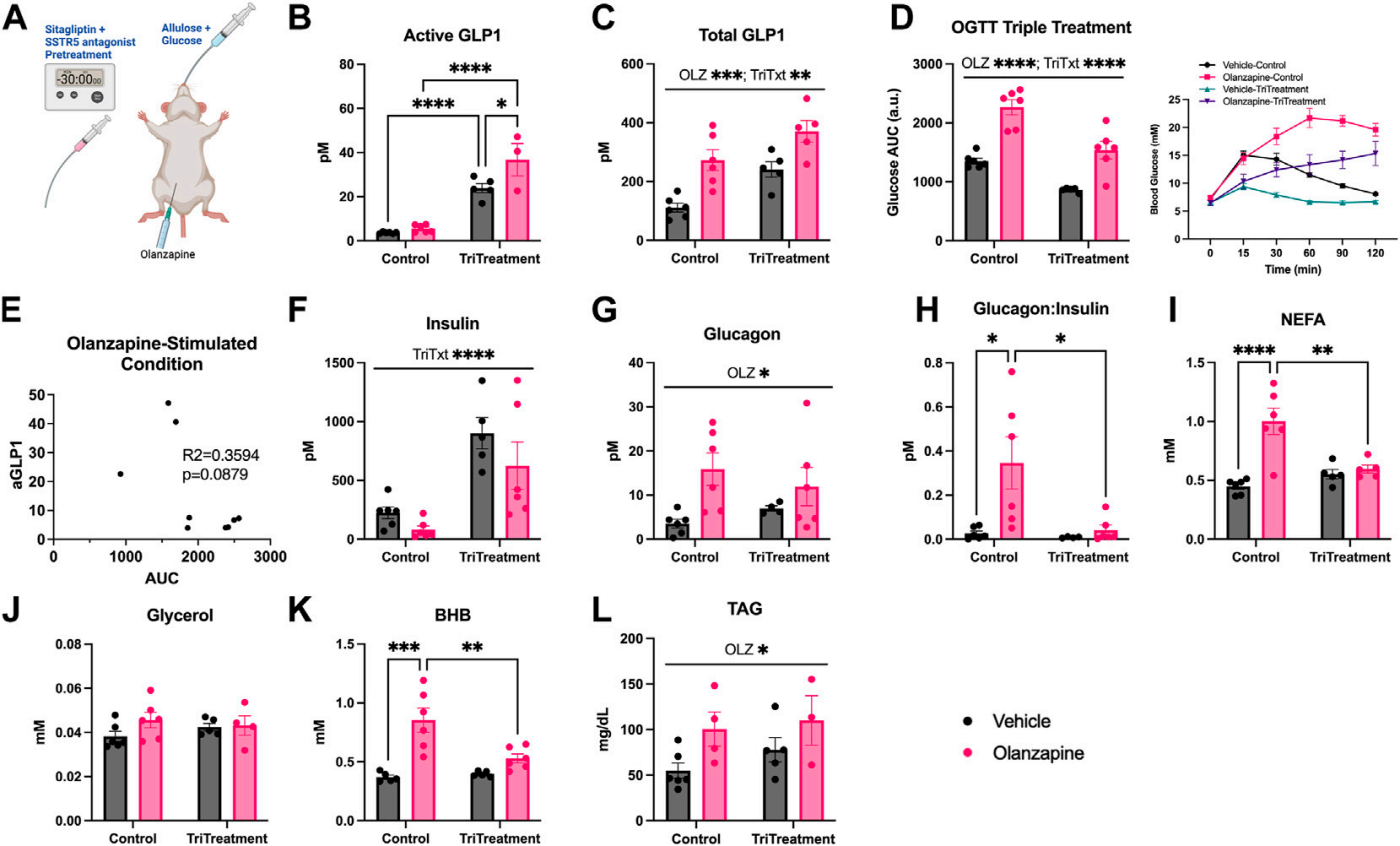
Triple treatment during OGTT improves olanzapine-induced changes in glucose and lipid homeostasis. Mice were treated with a mixed oral gavage of sitagliptin (10 mg/kg) and SSTR5 antagonist (15 mg/kg) 30 min prior to a mixed oral gavage of allulose (1 g/kg) and glucose (0.5 g/kg) and an IP injection of olanzapine (5 mg/kg) **(A)** (*n* = 4–6 mice/group). **(B, C)** Active and total GLP1 were measured from serum following 120 min from injection. **(D)** Blood glucose was measured from the distal tail blood and area under the curve (AUC) calculated. **(E)** A correlational analysis was performed between active GLP1 and blood glucose AUC in olanzapine-treated animals. In serum collected from cardiac blood insulin **(F)**, glucagon **(G)**, the ratio of glucagon:insulin **(H)**, Non-Esterified Fatty Acids **(I)**, glycerol **(J)**, Beta-Hydroxybutyrate **(K)**, and Triacylglycerol (**L**) were measured. Blood glucose AUC and serum hormones/metabolites were analyzed by two-way ANOVA. Lines over graphs indicate a main effect of the described parameter while bars connected by lines indicate a significant difference between indicated groups. *p < 0.05, **p < 0.01, ***p < 0.001, ****p < 0.0001. All data are presented as mean ± SEM.

Although triple treatment did not protect against olanzapine induced increases in blood glucose a caveat to this initial experiment was that it was completed in the absence of an additional homeostatic challenge. There is evidence that a nutrient stimulus may be necessary to observe acute AP-induced glucose dysregulation (Castellani et al., 2022), (Albaugh et al., 2011b). This is likely an important consideration given the impact of carbohydrate consumption on GLP1 secretion and the fact that individuals prescribed olanzapine could be consuming carbohydrate after taking their medication. In an effort to test the effects of triple treatment under perhaps more clinically relevant conditions we assessed the blood glucose response to olanzapine and/or triple treatment in mice following an oral gavage of glucose (0.5 g/kg) (Figure 4A). This glucose dose was chosen based on pilot experiments which showed that average glucose values of olanzapine treated animals fell within the detection range of the glucometer.

Triple treatment increased active GLP1 (Mean ± SD: Vehicle-Control = 3.64 ± 0.35, Olanzapine-Control = 5.59 ± 1.72, Vehicle-TriTreatment = 23.90 ± 4.67, Olanzapine-TriTreatment = 36.79 ± 12.70; Figure 4B) with the largest increase in animals that received triple treatment and olanzapine. There was a significant main effect of olanzapine and triple treatment to increase total GLP1 (Mean ± SD: Vehicle-Control = 111.25 ± 36.24, Olanzapine-Control = 272.84 ± 87.42, Vehicle-TriTreatment = 241.19 ± 57.95, Olanzapine-TriTreatment = 371.16 ± 82.25; Figure 4C). Blood glucose AUC was significantly increased by olanzapine and lower in animals given triple treatment (Figure 4D) and there was a trend (*p* = 0.0879) for the glucose AUC being negatively correlated with active GLP1 in olanzapine-treated animals (Figure 4E). Insulin was increased by triple treatment (Figure 4F) while glucagon was increased by olanzapine (Figure 4G) resulting in the increase in glucagon:insulin with olanzapine being absent in mice receiving triple treatment (Figure 4H).

Markers of perturbed lipid metabolism following olanzapine treatment such as increases in serum NEFA (Figure 4I) and BHB (Figure 4K), an index of whole-body fatty acid oxidation, were reduced with triple treatment. Conversely, serum TAG was elevated by olanzapine and unaffected by triple treatment (Figure 4L). In this experiment we provide evidence that increasing GLP1 to supraphysiological levels during a glucose challenge confers a degree of protection against acute olanzapine induced perturbations in glucose and lipid metabolism. There were no differences in serum levels of olanzapine and N-desmethyl-olanzapine (DMO) between control and triple treatment animals, suggesting that these protective effects are not secondary to differences in circulating drug concentrations (Table 1).

**TABLE 1:** Serum measures of olanzapine and N-desmethyl-olanzapine (DMO) taken 2 h following olanzapine or olanzapine plus triple treatment in male C57BL/6J mice. *p*-values for unpaired, 2-tailed t-tests are provided. *p* < 0.05 are considered statistically significant.

	Control	Triple	*p*-value
		Treatment	
Olanzapine (ng/mL)	124.81 ± 5.14	138.12 ± 5.69	*p* = 0.11
DMO (ng/mL)	31.26 ± 1.03	30.94 ± 2.95	*p* = 0.92

## Discussion

Treatment with antipsychotic drugs such as olanzapine can result in severe metabolic consequences such as hyperglycemia, dysregulated lipid metabolism, weight gain and the development of type 2 diabetes, in a population that suffers from premature cardiovascular mortality (Barton et al., 2020), (De Hert et al., 2009). An unappreciated aspect of antipsychotic drugs is the acute perturbations in glucose and lipid metabolism that occur independent of changes in body weight and adiposity (Shamshoum et al., 2019), [(Bush et al., 2018; Castellani et al., 2018; Shamshoum et al., 2021a)], (Albaugh et al., 2012), (Shamshoum et al., 2021b). In the current investigation we use agents which increase and sustain endogenous GLP1, these include allulose, sitagliptin, and an SSTR5 antagonist and demonstrate that, in combination, this cocktail offers a degree of protection against olanzapine-induced perturbations in glucose and lipid homeostasis which occurred independent of any changes in serum olanzapine concentrations.

GLP1 receptor agonists such as liraglutide have proven to be effective pharmacological tools to reduce weight gain in individuals treated with antipsychotics [(Sacher et al., 2008; Ikegami et al., 2013b; Larsen et al., 2017; Lee et al., 2021)]. In addition GLP1 receptor agonism also potently protects against acute olanzapine induced perturbations in carbohydrate and fat metabolism which correspond with reductions in the glucagon:insulin ratio (Medak et al., 2020). In the current study supraphysiological increases in circulating GLP1 offer a degree of protection against olanzapine-induced glucose tolerance and lipidemia, in parallel with a reduction in glucagon:insulin. Interestingly, this triple treatment did not have a protective effect without the metabolic challenge of oral glucose, as opposed to liraglutide, which did (Medak et al., 2020). The distinct response of our treatment with or without glucose could be related to the larger increase in total GLP1 with glucose but this is somewhat confounded by the fact that the active form of GLP1 was the same, at least when measured after 2 h. It could be that peak increases in circulating active GLP1 were missed in the current study, however this seems somewhat unlikely as a similar triple treatment approach in mice has been reported to lead to consistent and sustained increases in active GLP1 for upwards of 2 hours (Briere et al., 2018). SSTR5 antagonist alone is effective at lowering blood glucose when glucose is delivered orally but not intraperitoneally (Jepsen et al., 2021), which is in line with our findings that triple treatment protects against olanzapine-induced glucose intolerance but not olanzapine-induced hyperglycemia. We did not evaluate SSTR5 antagonist as a co-treatment with olanzapine as this intervention is most effective in synergy with other interventions such as DPPiv inhibition (Liu et al., 2018b). Moderate increases in total and/or active GLP1 did not confer protection against olanzapine-induced hyperglycemia. We reasoned that combining triple treatment, the approach which caused the largest increase in GLP1, with an oral glucose challenge, which should further increase GLP1, could potentially uncover a protective effect of increased endogenous GLP1 against olanzapine-induced perturbations in glucose homeostasis. While this proved to be true it should be noted that the relative increase in blood glucose between control and “triple treated” mice appeared to be similar. Moving forward, it will be important to determine if similar protective effects are noted with mono or dual treatment approaches under glucose stimulated conditions.

A potential confounder to the current experiments is that we cannot account for other hormones that might be stimulated by oral glucose and preserved by inhibition of DPPiv such as gastric inhibitory peptide (GIP), an incretin hormone and substrate of DPPiv (Deacon et al., 2001). We surmise that action by GIP is not the main mediator of our protective effect on olanzapine-induced metabolic dysfunction because metabolically compromised individuals have a reduced insulinotropic response to GIP but not GLP1 (Nauck et al., 1993), (Mentis et al., 2011) and GIP is a stimulator of glucagon secretion even during hyperglycemia in individuals with type 2 diabetes (Mentis et al., 2011). To further support this, GIP receptor null mice treated with a similar GLP1 enhancing cocktail displayed marked improvements in oral glucose tolerance (Briere et al., 2018) providing good evidence that the protective effects of the triple treatment were not mediated by GIP.

Treatment with GLP1 receptor agonists are effective and generally well-tolerated but they can be limited by the requirement of repeated injections, dose escalation, nausea, and injection-site reactions (Harris and McCarty, 2015). By treating orally with a cocktail of agents that includes a highly palatable sugar we circumvent these possible complications. From a clinical perspective, treatment with agents which increase endogenous GLP1 could be effective against olanzapine-induced metabolic disturbances in patients when exogenous glucose is ingested, and this is important as AP-treated patients display increased consumption of refined sugar products (Blouin et al., 2008). Future work would be required to determine how often triple treatment should be administered as sitagliptin has a much longer half-life in human (∼12 h) than in rodents (1–2 h) (Kim et al., 2005), (Zerilli and Pyon, 2007). It is not clear whether these supraphysiological increases in GLP1 secretion can be sustained over time as our studies are limited to acute responses, though evidence from metabolic surgery suggests the durability of GLP1 output that is sustained for years in human patients (Briere et al., 2018), (Hutch and Sandoval, 2017).

Findings from the current investigation provide proof of principle that increasing endogenous levels of GLP1 can provide therapeutic benefit in alleviating the metabolic side effects of olanzapine. This ‘triple treatment’ targets complementary mechanisms to increase both active and total GLP1 and protect against olanzapine-induced perturbations in lipid and glucose metabolism, under glucose stimulated conditions. Besides the triple treatment in the current investigation there are other small molecules GPR40, GPR119, and TGR5, for example, that are not yet approved for human use which might also be useful in similar combination therapies to increase endogenous GLP1 to therapeutic levels (Briere et al., 2018), (Gimeno et al., 2020). Future translational research should consider manipulating circulating GLP1 as an adjunct treatment approach to lessen the acute metabolic consequences of APs in those with schizophrenia or other forms of severe mental illness.

## Data Availability

The raw data supporting the conclusion of this article will be made available by the authors, without undue reservation.
